# A Ca^2+^-stimulated exosome release pathway in cancer cells is regulated by Munc13-4

**DOI:** 10.1083/jcb.201710132

**Published:** 2018-08-06

**Authors:** Scott W. Messenger, Sang Su Woo, Zhongze Sun, Thomas F.J. Martin

**Affiliations:** Department of Biochemistry University of Wisconsin-Madison, Madison, WI

## Abstract

Cancer cells secrete copious amounts of exosomes, and elevated intracellular Ca^2+^ is critical for tumor progression and metastasis, but the underlying cellular mechanisms are unknown. Munc13-4 is a Ca^2+^-dependent SNAP receptor– and Rab-binding protein required for Ca^2+^-dependent membrane fusion. Here we show that acute elevation of Ca^2+^ in cancer cells stimulated a fivefold increase in CD63^+^, CD9^+^, and ALIX^+^ exosome release that was eliminated by Munc13-4 knockdown and not restored by Ca^2+^ binding–deficient Munc13-4 mutants. Direct imaging of CD63-pHluorin exosome release confirmed its Munc13-4 dependence. Depletion of Munc13-4 in highly aggressive breast carcinoma MDA-MB-231 cells reduced the size of CD63^+^ multivesicular bodies (MVBs), indicating a role for Munc13-4 in MVB maturation. Munc13-4 used a Rab11-dependent trafficking pathway to generate MVBs competent for exosome release. Membrane type 1 matrix metalloproteinase trafficking to MVBs by a Rab11-dependent pathway was also Munc13-4 dependent, and Munc13-4 depletion reduced extracellular matrix degradation. These studies identify a novel Ca^2+^- and Munc13-4-dependent pathway that underlies increased exosome release by cancer cells.

## Abstract (precis)

Messenger et al. show that exosome secretion in cancer cells is Ca^2+^-stimulated and dependent on Ca^2+^-bound Munc13-4. Munc13-4, a late endosome priming factor, acts via a Rab11-dependent pathway to prepare multivesicular endosomes for exocytosis. These results indicate that Munc13-4 plays a central role in exosome release in cancer cells.

## Introduction

Cytosolic Ca^2+^ levels control several signaling processes in normal cellular homeostasis. Disruption of normal Ca^2+^ is hypothesized to be a cause of enhanced proliferation and metastasis found in various cancers (Lee et al., 2011; Prevarskaya et al., 2011; Monteith et al., 2012; Déliot and Constantin, 2015). Store-operated calcium entry and the transient receptor potential channel family are amplified in many cancers to produce elevated Ca^2+^, although the exact genes responsible are cancer subtype specific (Lee et al., 2011; Prevarskaya et al., 2011; Monteith et al., 2012; Déliot and Constantin, 2015). Ca^2+^-dependent proliferation is mediated by MAPK/calmodulin-dependent pathways, whereas invasion and migration are enhanced via Ca^2+^-dependent cytoskeleton rearrangement and focal adhesion disassembly (Lee et al., 2011; Prevarskaya et al., 2011; Monteith et al., 2012; Déliot and Constantin, 2015). Although numerous studies have identified Ca^2+^ channels that are amplified with pathological consequences, roles for Ca^2+^-dependent effectors are poorly understood.

Exosomes are a class of extracellular vesicles 30–150 nm in diameter corresponding to intraluminal vesicles (ILVs) released by multivesicular body (MVB) exocytosis. Proteins such as the tetraspanin protein CD63 are characteristically found on exosomes but are absent from other extracellular vesicles (Mathivanan et al., 2012; Momen-Heravi et al., 2013). Exosomes contribute to cancer growth and metastasis through mechanisms that include transfer of oncogenes for enhanced proliferation (de Gassart et al., 2004; Kharaziha et al., 2012; Abels and Breakefield, 2016; Fu et al., 2016; Kalluri, 2016), extracellular matrix reorganization for migration and invasion (Hoshino et al., 2013; Sung et al., 2015; Becker et al., 2016; Sinha et al., 2016), and altered immune cell responses for impaired immune system surveillance (Liu et al., 2006; Clayton et al., 2007, 2008; Bobrie et al., 2011; Filipazzi et al., 2012). Given the multiple roles of exosomes in cancer progression, understanding the cellular basis of exosome release is critically important.

Several ESCRT and related proteins including HRS, STAM1, TSG101 (Colombo et al., 2013), ALIX (Baietti et al., 2012), and VPS4 (Jackson et al., 2017) have been implicated in exosome release; however, it is unclear if ESCRTs are acting on the plasma membrane to evaginate extracellular vesicles or in ILV formation on MVBs to regulate exosome release (Kowal et al., 2014; Vader et al., 2014; Abels and Breakefield, 2016). Members of the Rab GTPase family such as Rab2, 5, 11, 27a, 27b, and 35 have also been found to regulate exosome release (Savina et al., 2002; Hsu et al., 2010; Ostrowski et al., 2010). Rab27a tethers MVBs near the plasma membrane, and its depletion prevents exosome release in numerous cancer cell lines (Ostrowski et al., 2010; Webber et al., 2010, 2015; Bobrie et al., 2012b; Li et al., 2014) but the role of the other Rabs is less clear. Moreover, the regulatory steps and involvement of acute Ca^2+^ elevation remain to be identified.

Munc13-4 is a Ca^2+^-dependent Rab binding protein characterized for its role in granule exocytosis in cytotoxic T lymphocytes (CTLs). Patients with familial hemophagocytic lymphohistiocytosis 3 (FHL3) have loss-of-function mutations in Munc13-4 resulting in cytotoxic granules that dock at the plasma membrane but fail to fuse, leading to deficiencies in target cell killing (Feldmann et al., 2003). Munc13-4 contains N- and C-terminal Ca^2+^-binding C2 domains, and mutations in Ca^2+^-binding C2 domain residues prevent Ca^2+^-dependent interactions of Munc13-4 with SNARE proteins and phospholipids (Boswell et al., 2012; Chicka et al., 2016; He et al., 2016). RBL-2H3 basophilic leukemia cells depleted for Munc13-4 exhibit reduced Ca^2+^-dependent secretory granule exocytosis that is restored by wild-type but not by Ca^2+^ binding–deficient Munc13-4 (Boswell et al., 2012; Woo et al., 2017). Ca^2+^ stimulation of exosome release was previously reported (Vincent-Schneider et al., 2001; Savina et al., 2002, 2003, 2005; Fauré et al., 2006); however, major Ca^2+^-regulated steps have yet to be characterized. The current work identified Munc13-4 as a major Ca^2+^-dependent regulator of a Rab11-dependent trafficking pathway to MVBs that was increased in cancer cells. This Ca^2+^-, Munc13-4–, and Rab11-dependent pathway generated secretion-competent MVBs for basal and Ca^2+^-stimulated CD63^+^ exosome release. We propose that the increased expression of Munc13-4 in cancer cells after epithelial-to-mesenchymal transition combined with increased Ca^2+^ uptake (Yang et al., 2009; Feng et al., 2010; Prevarskaya et al., 2011; Sun et al., 2014; Didiasova et al., 2015) drives enhanced exosome release by highly aggressive cancer cells, and that Munc13-4 is a potential target for therapeutic intervention.

## Results

### Exosome release is Ca^2+^ stimulated and controlled by Munc13-4

**Figure 1.**
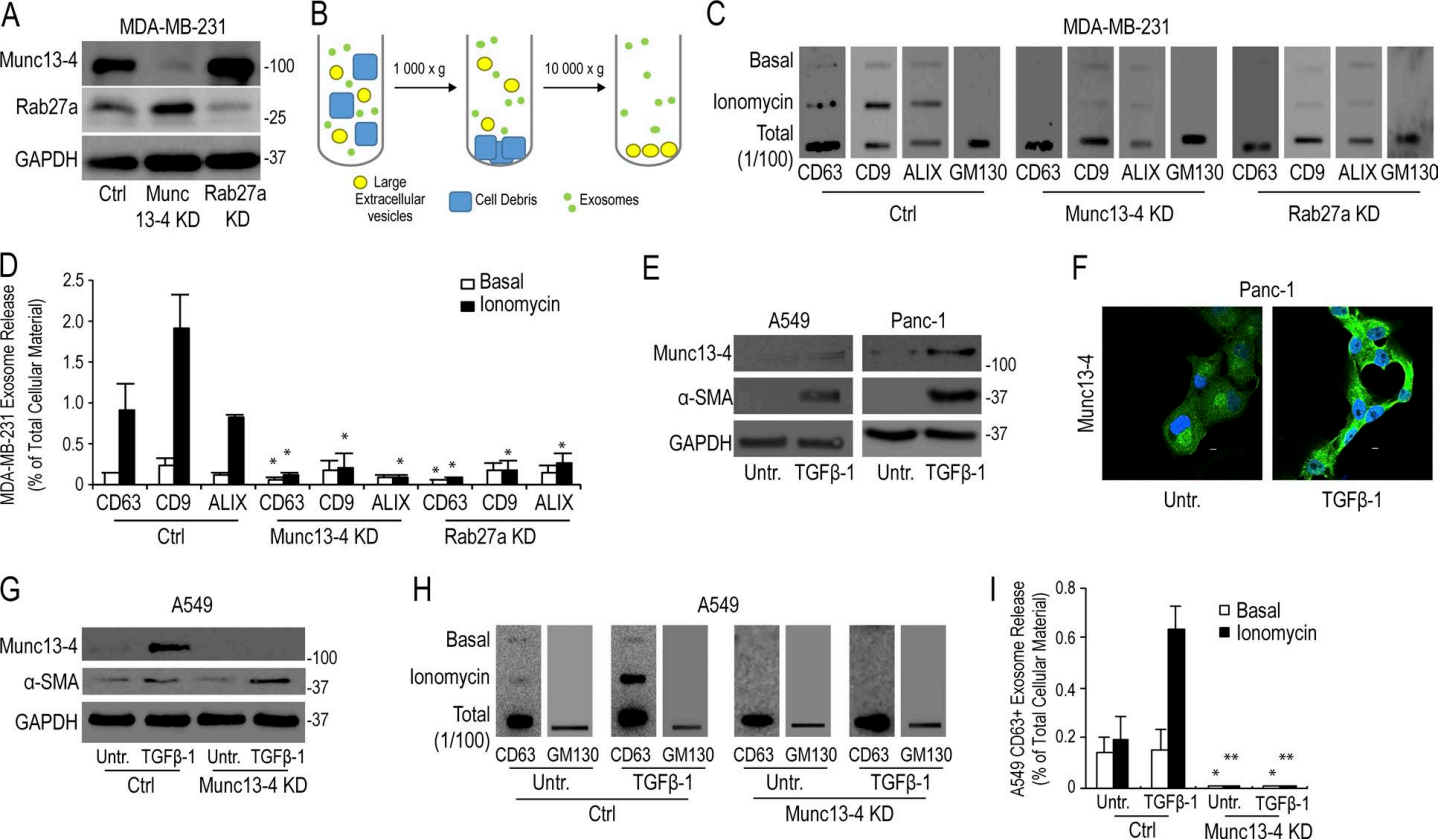
**Munc13-4 KD strongly impairs exosome release. (A)** SDS-PAGE Western blot of indicated proteins in MDA-MB-231 cells after stable expression of shRNA for Munc13-4 or Rab27a or a scrambled control (Ctrl). **(B)** Culture medium from MDA-MB-231 cells either untreated or stimulated with 1.25 µM ionomycin for 30 min was centrifuged at 1,000 *g* to remove cellular debris and 10,000 *g* to remove large extracellular vesicles. **(C)** The resulting 10,000-*g* supernatant was filtered onto a nitrocellulose membrane and analyzed for CD63, CD9, ALIX, and GM130 content by antibody blotting. **(D)** Quantification of CD63, CD9, and ALIX blots in C are shown as exosome release as a percentage of total cellular material with mean values ± standard error (SE) for *n* ≥ 3. *, P < 0.05 for comparison with corresponding control samples. **(E)** Panc-1 or A549 cells were left untreated or were treated with TGFβ-1 for 24 h. Indicated proteins were detected by SDS-PAGE Western blot. **(F)** Panc-1 cells were left untreated or were treated with TGFβ-1 for 24 h, and Munc13-4 levels were determined by immunofluorescence. TGFβ-1–treated cells exhibited a mesenchymal morphology. Bars, 5 μm. **(G)** A549 cells stably expressing control shRNA (Ctrl) or Munc13-4 shRNA were left untreated (Untr) or were treated with TGFβ-1 for 24 h, and SDS-PAGE Western blotting for indicated proteins was conducted. **(H)** Culture media supernatants (as in B) from A549 cells that were either untreated or were stimulated with 1.25 µM ionomycin for 30 min were filtered onto nitrocellulose membrane and analyzed for CD63 and GM130. **(I)** Quantification of CD63^+^ exosome release shown as a percentage of total cellular material with mean values ± SE for *n* = 5. *, P < 0.05; **, P < 0.01 for comparison with corresponding control samples.

The basis for enhanced exosome release by cancer cells is not understood, and regulatory proteins responsible for increased exosome secretion have not been identified. We initially focused on MDA-MB-231 cells, highly aggressive breast carcinoma cell lines commonly used for exosome release studies. Most studies of exosome release fail to take into account possible changes in cellular MVB/ILV pools. To quantitate exosome release as the percentage of cellular pools, we used a filter binding assay. Culture media containing secreted exosomes from cells were centrifuged at 1,000 *g* to remove cellular debris and at 10,000 *g* to clear large shed membranes and apoptotic extracellular vesicles (Fig. 1 B). The resulting supernatant was filtered onto a membrane in parallel with 1% of total cellular material to obtain exosome release as the percentage of total cellular material.

The acute (30-min) elevation of intracellular Ca^2+^ with ionomycin treatment enhanced CD63^+^, CD9^+^, and ALIX^+^ exosome release by approximately fivefold in control MDA-MB-231 cells (Fig. 1, C and D). Ca^2+^ stimulation increased exosome secretion from low resting levels of ∼0.1–0.2% to ∼1–2% in 30-min incubations. Exosome release as percentage of total is likely underestimated, in part because of exosome tethering to membranes (Edgar et al., 2016; see Discussion). We tested roles for Rab27a, a known regulator of exosome release (Ostrowski et al., 2010), and Munc13-4, one of the potential effectors of Rab27, by reducing protein levels of either by >95% with lentiviral shRNA knockdown (KD; Fig. 1 A). Munc13-4 KD completely eliminated Ca^2+^-stimulated exosome release and significantly reduced basal CD63^+^ exosome release but left basal CD9 and ALIX release intact (Fig. 1, C and D). Rab27a KD similarly inhibited exosome release. In all groups, the release of the Golgi membrane protein GM130 was <0.1% of total cellular Golgi GM130, indicating that little cell lysis or apoptosis occurred under these stimulation conditions.

Established methods for detecting secreted exosomes, including density enrichment, dynamic light scattering, electron microscopy, and protein gel staining, produced the same results as the quantitative filter blotting assay (Fig. S1, A–F), which confirmed that Ca^2+^-stimulated exosome release is regulated by Munc13-4. CD63 is found exclusively on exosomes and is used to distinguish exosomes from other extracellular vesicles (Bobrie et al., 2012a; Edgar et al., 2014; Kowal et al., 2014, 2016; Tkach and Théry, 2016). Therefore, CD63 was used as the primary marker for exosomes in subsequent experiments. These findings indicate that Munc13-4 is a positive regulator of Ca^2+^-stimulated CD63^+^ exosome release, apparently operating on a subset of CD63^+^ MVBs.

### Up-regulation of Munc13-4 enhances Ca^2+^-stimulated exosome release

TGFβ-1 induces a more aggressive in vitro phenotype in certain cancer cell lines, including the human lung carcinoma line A549 and the human pancreatic carcinoma line Panc-1. Microarray screens showed that Munc13-4 mRNA was elevated after 24-h TGFβ-1 treatment in A549 cells (Sartor et al., 2010) and Panc-1 cells (Maupin et al., 2010). To confirm that Munc13-4 protein levels were also elevated, A549 and Panc-1 cells were either untreated or stimulated with 5 ng/ml TGFβ-1 for 24 h. Gel immunoblot analysis showed that 120-kD Munc13-4 protein levels were markedly increased, as were levels of the TGFβ-1–responsive gene α-smooth muscle actin (Fig. 1 E). A similar increase in Munc13-4 protein by TGFβ-1 treatment was detected in Panc-1 cells, where the acquisition of mesenchymal morphology was also found (Fig. 1 F). In untreated A549 cells, Ca^2+^ stimulation produced no effect on CD63^+^ exosome release, whereas Ca^2+^ stimulation induced a threefold increase in CD63^+^ exosome release in TGFβ-1–treated A549 cells (Fig. 1, G–I). Munc13-4 KD fully ablated both basal and Ca^2+^-stimulated CD63^+^ exosome release in TGFβ-1–treated A549 cells. A similar increase in Ca^2+^ responsiveness and Munc13-4 dependence was observed in Panc-1 cells after TGFβ-1 treatment, although untreated Panc-1 cells exhibited Ca^2+^-dependent exosome release as a result of increased basal Munc13-4 levels (Fig. S2, A–C). It should be noted that Munc13-4 levels in TGFβ-1–treated Panc-1 cells were comparable to those in the highly aggressive MDA-MB-231 cells (Fig. S2 D). Collectively, the results show that increased Munc13-4 expression is associated with Ca^2+^-stimulated, Munc13-4–dependent exosome release, which provides an explanation for enhanced exosome secretion in metastatic cancer cells.

### Ca^2+^-dependent Munc13-4 membrane association is required for exosome release

**Figure 2.**
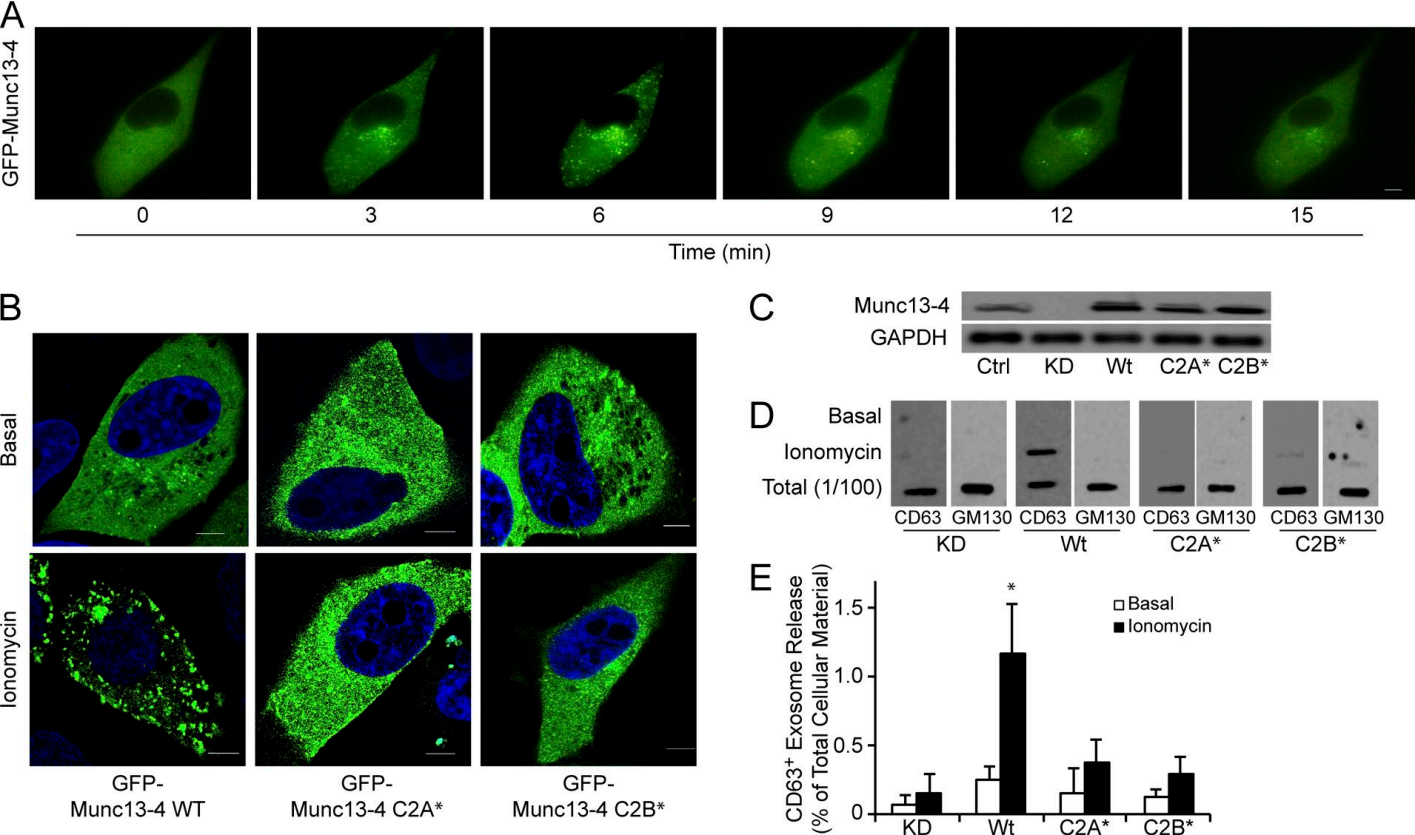
**Munc13-4 translocation to membrane is Ca^2+^ dependent. (A)** Live-cell epifluorescence imaging of GFP-Munc13-4 in MDA-MB-231 cells at indicated times after ionomycin stimulation. See Video 1. **(B)** MDA-MB-231 cells expressing wild-type GFP-Munc13-4, GFP-Munc13-4 C2A*, or GFP-Munc13-4 C2B* either left untreated or stimulated with 1.25 µM ionomycin for 5 min were fixed and imaged by confocal microscopy. **(C)** Indicated proteins were detected by SDS-PAGE and Western blotting of lysates with Munc13-4 antibody from MDA-MB-231 cells stably expressing control shRNA (Ctrl) or shRNA targeting Munc13-4 (KD), or Munc13-4 KD cells rescued with shRNA-resistant wild-type Munc13-4, Munc13-4 C2A*, or Munc13-4 C2B*. **(D)** Culture media supernatants (as in Fig. 1 B) from MDA-MB-231 cells as in C either untreated or stimulated with 1.25 µM ionomycin for 30 min were filtered onto membrane and analyzed for CD63 or GM130. **(E)** Quantification of CD63^+^ exosome release (from Fig 1 D) shown as mean values ± SE for *n* = 3. *, P < 0.05 for comparison between ionomycin-treated and basal. Bars, 5 μm.

We previously reported that Ca^2+^ regulates Munc13-4 activity (Boswell et al., 2012). Mutations of the two Ca^2+^-binding aspartates to asparagines (D127N and D133N) in the C2A domain (Munc13-4 C2A*) inhibited Ca^2+^-dependent interactions of Munc13-4 with SNARE proteins, whereas similar mutations in the C2B domain (D941N, D947N; Munc13-4 C2B*) prevented Ca^2+^-stimulated membrane binding (Boswell et al., 2012; Chicka et al., 2016). In resting MDA-MB-231 cells, GFP-Munc13-4 was dispersed in the cytoplasm with no apparent membrane localization (Fig. 2 A). However, Ca^2+^-elevations recruited GFP-Munc13-4 to punctate structures maximally at 3–5 min before slow dissociation over the next 20 min (Fig. 2 A and Video 1). A greater proportion of Munc13-4 was membrane associated after Ca^2+^ stimulation when MDA-MB-231 cells were fixed, which is likely a result of the rapid association/dissociation of Munc13-4 binding to membrane (Figs. 2 B and 4 A). In accord with the Ca^2+^-dependence of Munc13-4, Ca^2+^-stimulated membrane recruitment was reduced for GFP-Munc13-4 C2A* and eliminated for GFP-Munc13-4 C2B* (Fig. 2 B). To determine the correspondence of membrane association and activity for Munc13-4, we tested the ability of mutant Munc13-4 proteins to rescue Ca^2+^-stimulated exosome release in Munc13-4 KD MDA-MB-231 cells. shRNA-resistant wild-type Munc13-4 fully rescued, whereas shRNA-resistant Munc13-4 C2A* or Munc13-4 C2B* failed to rescue, Ca^2+^-stimulated CD63^+^ exosome release in Munc13-4 KD cells (Fig. 2, C–E). The results suggest that the Ca^2+^-dependent recruitment of Munc13-4 to membrane is required for Ca^2+^-stimulated exosome release.

### Munc13-4 regulates MVB maturation

**Figure 3.**
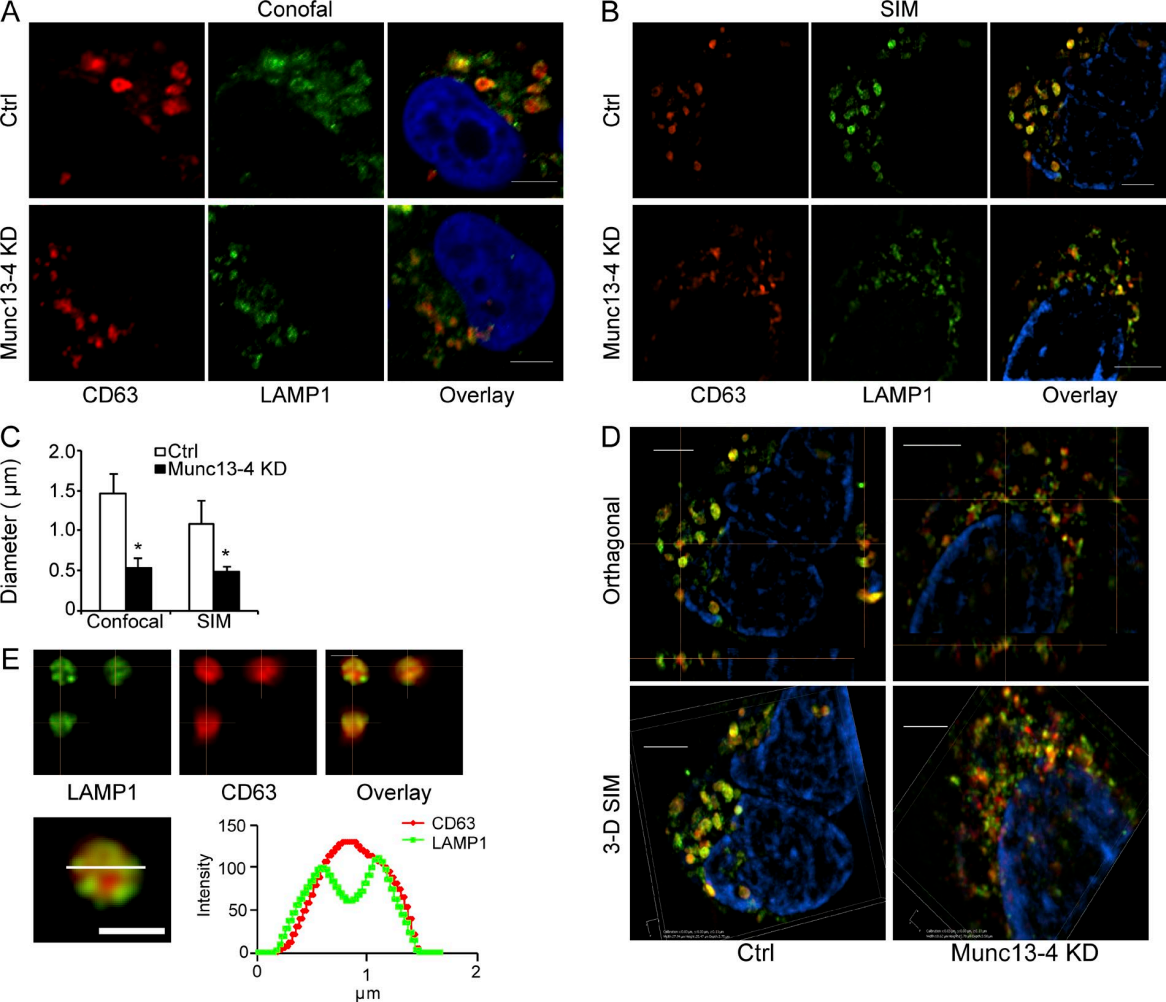
**Munc13-4 regulates MVB size. (A and B)** MDA-MB-231 cells stably expressing control shRNA (Ctrl) or Munc13-4 shRNA were fixed for immunofluorescence detection of endogenous CD63 (red) and LAMP1 (green) by confocal microscopy (A) or SIM (B). Bars, 5 μm. **(C)** Mean diameter of CD63^+^ structures in each indicated cell type imaged by confocal or SIM shown as mean values ± SE for three cells/group from three separate preparations (*, P < 0.05). **(D)** SIM showing orthogonal (top) and 3D reconstruction view of CD63 (red) and LAMP1 (green) in MDA-MB-231 cells stably expressing control shRNA (Ctrl) or Munc13-4. Bars, 5 μm. **(E)** 3D reconstruction of SIM images from Ctrl MDA-MB-231 cell in D of a single MVB labeling CD63 (red) and LAMP1 (green). Linescan of indicated channels is shown on the bottom. Bars, 1 µm.

Munc13-4 regulates aspects of late endosomal fusion in neutrophils (He et al., 2016) and mediates Ca^2+^-stimulated homotypic late endosome fusion in RBL-2H3 cells to generate an enlarged endosomal vacuole that is CD63^+^ (Woo et al., 2017). MDA-MB-231 cells contain CD63^+^ and LAMP1^+^ MVBs that reach >1 µm in diameter as imaged by confocal and structured illumination microscopy (SIM; Fig. 3, A and B). We found that KD of Munc13-4 resulted in a reduced mean size of the CD63^+^ MVBs from 1.49 ± 0.24 µm to 0.54 ± 0.11 µm by confocal microscopy and from 1.07 ± 0.30 to 0.48 ± 0.07 µm by SIM (Fig. 3 C). Rab27a KD had no effect on MVB size (not depicted), which is consistent with its proposed role in MVB exocytosis (Ostrowski et al., 2010) rather than MVB maturation. 3D SIM revealed that CD63 was present on MVBs, as was LAMP1 (Fig. 3 D). Linescans through the equatorial plane of individual MVBs indicated that CD63 was present in the core of MVBs, possibly as unresolved ILVs, as well as at the limiting membrane (Fig. 3 E). In contrast, linescans showed two peaks for LAMP1, suggesting it is enriched on the MVB membrane. Overall, Munc13-4 regulated MVB size, as previously reported for RBL-2H3 cells (Woo et al., 2017).

### Munc13-4 functions in a Rab11a trafficking pathway to regulate exosome release

**Figure 4.**
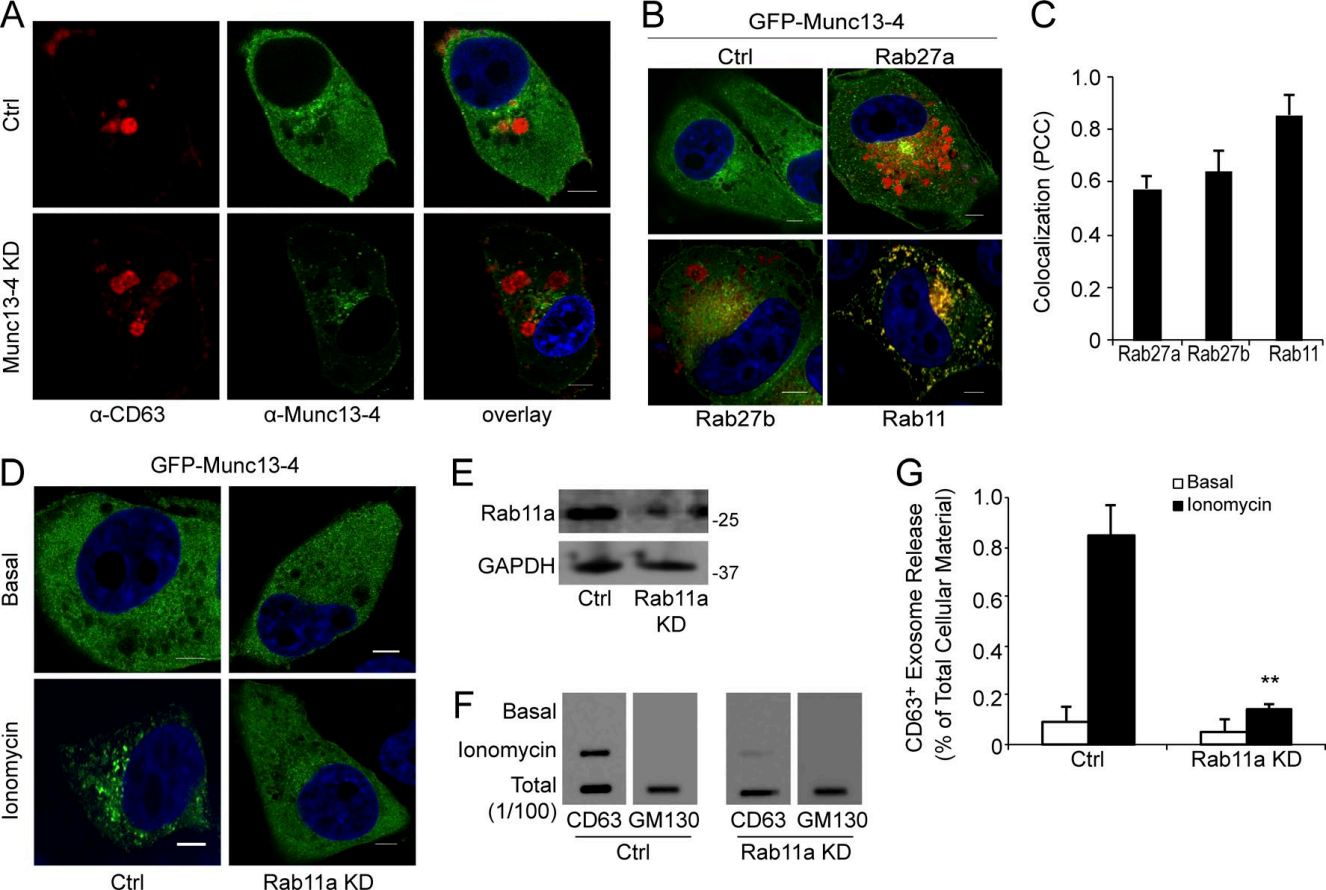
**Munc13-4 recruitment to recycling endosomes is dependent on Rab11a. (A)** MDA-MB-231 cells immunolabeled for endogenous CD63 (red) and Munc13-4 (green) by confocal microscopy. **(B)** MDA-MB-231 cells expressing GFP-Munc13-4 and mCherry-Rab27a, mCherry-Rab27b, or mApple-Rab11a imaged by confocal microscopy. **(C)** Percentage of cells with Pearson’s correlation coefficient for GFP versus mCherry/mApple >0.7. **(D)** MDA-MB-231 cells stably expressing control shRNA (Ctrl) or Rab11a shRNA and GFP-Munc13-4 were left untreated or were stimulated with 1.25 µM ionomycin for 5 min, fixed, and imaged by confocal microscopy. **(E)** SDS-PAGE Western blot of indicated proteins in cells stably expressing control shRNA (Ctrl) or Rab11a shRNA. **(F)** Culture media supernatants (as in Fig. 1 B) from control (Ctrl) or MDA-MB-231 cells stimulated with 1.25 µM ionomycin for 30 min were filtered onto membrane and immunoblotted for CD63 and GM130. **(G)** Quantification of CD63^+^ exosome release as percentage of cellular total indicated as mean values ± SE for *n* = 5. **, P < 0.01 for comparison of ionomycin-treated samples. Bars, 5 μm.

Although Munc13-4 is required for CD63^+^ exosome release, endogenous Munc13-4 showed limited if any recruitment to CD63^+^ structures after Ca^2+^-stimulation (Fig. 4 A). Instead, we found that Munc13-4 was recruited to Rab11^+^ structures upon Ca^2+^ stimulation rather than to Rab27a^+^ or Rab27b^+^ structures (Fig. 4, B and C). Consistent with this, Munc13-4 was reported to bind Rab11 (Johnson et al., 2016) and was implicated in the merging of Rab11^+^/Munc13-4^+^ endosomes with late endosomes in CTLs (Ménager et al., 2007). Given the recruitment of Munc13-4 to Rab11a^+^ structures, we investigated a role for Rab11a in Munc13-4–dependent exosome release. The KD of Rab11a was found to prevent Ca^2+^-stimulated GFP-Munc13-4 membrane recruitment (Fig. 4 D) and strongly inhibit Ca^2+^-stimulated exosome release in MDA-MB-231 cells (Fig. 4, E–G). The results indicate that Munc13-4 functions in Ca^2+^-stimulated exosome release through a Rab11-dependent trafficking pathway.

**Figure 5.**
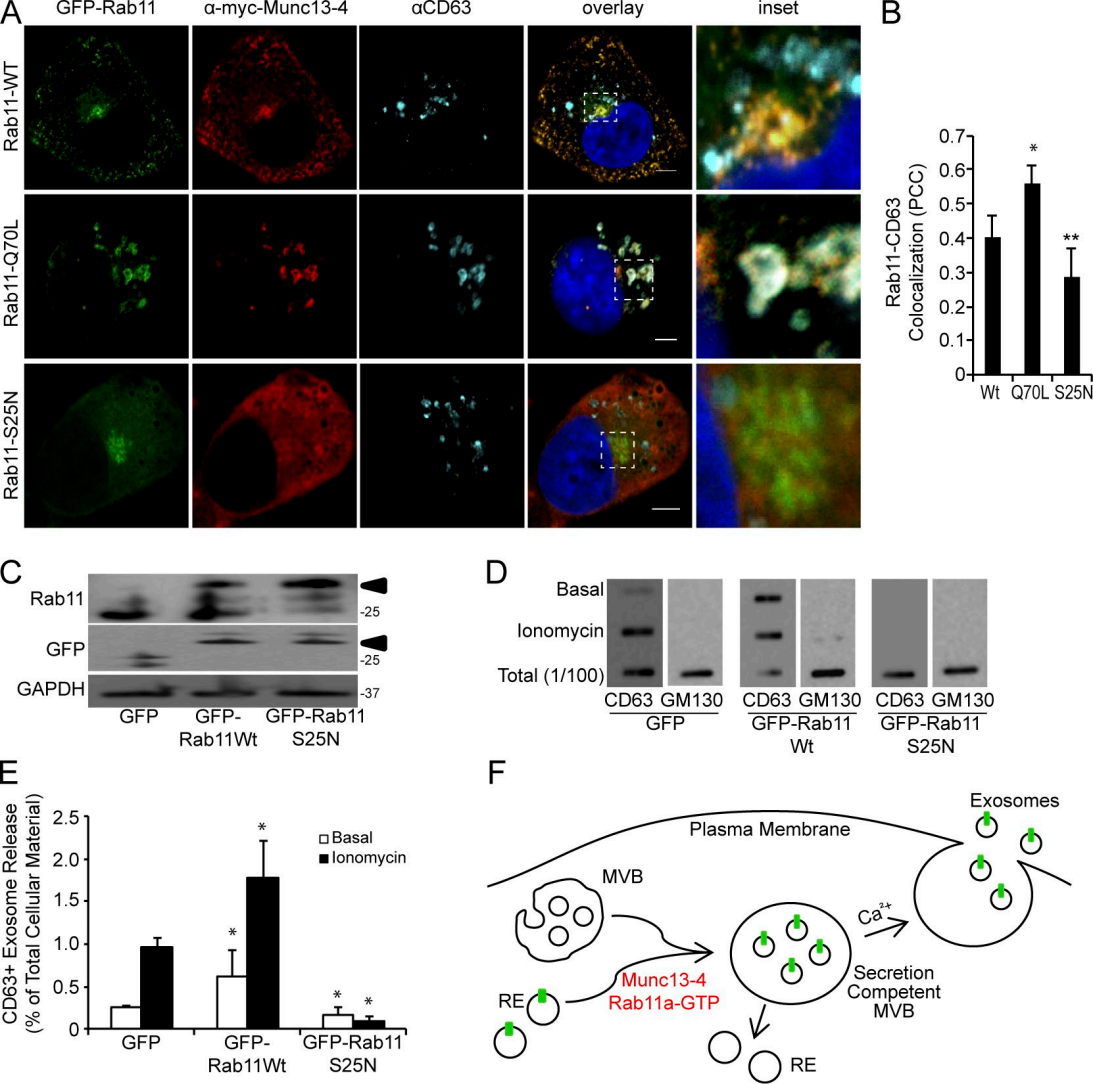
**Munc13-4 function in exosome release is dependent on Rab11a. (A)** MDA-MB-231 cells expressing GFP-Rab11, GFP-Rab11 Q70L, or GFP-Rab11 S25N were immunolabeled for myc-Munc13-4 (red) and endogenous CD63 (cyan). Bar, 5 µm. **(B)** Pearson’s correlation coefficient for GFP-Rab11 versus CD63 immunolocalization. Mean values ± SE (15 cells/group from three separate preparations) are shown. *, P < 0.05; **, P < 0.01. **(C)** MDA-MB-231 cells expressing GFP, GFP-Rab11, or GFP-Rab11 S25N were immunoblotted for the indicated proteins. Arrow indicates GFP-Rab11; lower band in upper panel corresponds to Rab11. **(D)** Cell medium supernatants (as in Fig. 1 B) from untreated MDA-MB-231 cells or cells treated with 1.25 µM ionomycin were filtered onto membrane and analyzed for CD63 and GM130. **(E)** Quantification of CD63 exosome release as percentage of total cellular shown as mean values ± SE (*n* = 3) with *, P < 0.05 for comparison to GFP-alone samples. **(F)** Model for the generation of secretion-competent MVBs as arising from the transient fusion of Rab11^+^ endosomes with MVB precursors, which requires Munc13-4, Ca^2+^, and Rab11a. MT1-MMP (green bar) is found in the recycling endosome (RE) and delivered to the MVB dependent on Munc13-4 for incorporation to ILVs and exosome release. The secretion-competent MVBs generated acquire components for Ca^2+^-dependent fusion with the plasma membrane. Bars, 5 μm.

Although Rab11a appeared to regulate CD63^+^ exosome release in MDA-MB-231 cells, Rab11a showed very limited colocalization (Pearson’s correlation coefficient <0.4) with CD63 in these cells (Fig. 5, A and B). However, expression of a constitutively active GFP-Rab11a Q70L mutant resulted in both Rab11a and Munc13-4 colocalizing at the limiting membrane of the CD63^+^ MVBs, which significantly increased the Pearson’s correlation coefficient to 0.55 (Fig. 5, A and B). Conversely, expression of a GFP-Rab11a S25N dominant negative mutant prevented Munc13-4 membrane recruitment and decreased the size of CD63^+^ structures (Fig. 5 A), which is similar to the effect of Munc13-4 KD (Fig. 3). Collectively, the results indicate that wild-type Rab11a and Munc13-4 traffic to MVBs but only transiently associate with the MVBs.

Finally, we assessed whether Rab11a-dependent trafficking to MVBs was an important determinant for exosome release. Overexpression of wild-type GFP-Rab11 enhanced both basal and Ca^2+^-stimulated CD63^+^ exosome release by ∼60%, whereas overexpression of the dominant negative Rab11a-S25N decreased Ca^2+^-stimulated exosome release by >90% (Fig. 5, D and E). Overexpression of GFP-Rab11aS25N was also found to deplete endogenous Rab11a levels (Fig. 5 C) as reported (Messenger et al., 2015). These results indicate the importance of Rab11a for exosome release as reported for erythroleukemia cells (Savina et al., 2002, 2003, 2005). Collectively, our findings indicate that GTP-bound Rab11a enhances trafficking of Rab11a^+^/Munc13-4^+^ endosomes to MVBs to promote a size increase and secretion competence. Wild-type Rab11a and Munc13-4 only transiently associate with the MVBs, dependent on the GTP cycle of Rab11a. Together with the finding that Munc13-4 KD decreases the size of MVBs and strongly impairs MVB secretion competence, these studies indicate that there is a Ca^2+^-, Munc13-4–, and Rab11a-dependent endosomal trafficking pathway that generates secretion-competent MVBs for Ca^2+^-stimulated exosome release (Fig. 5 F).

### Imaging exosome release in live cells

**Figure 6.**
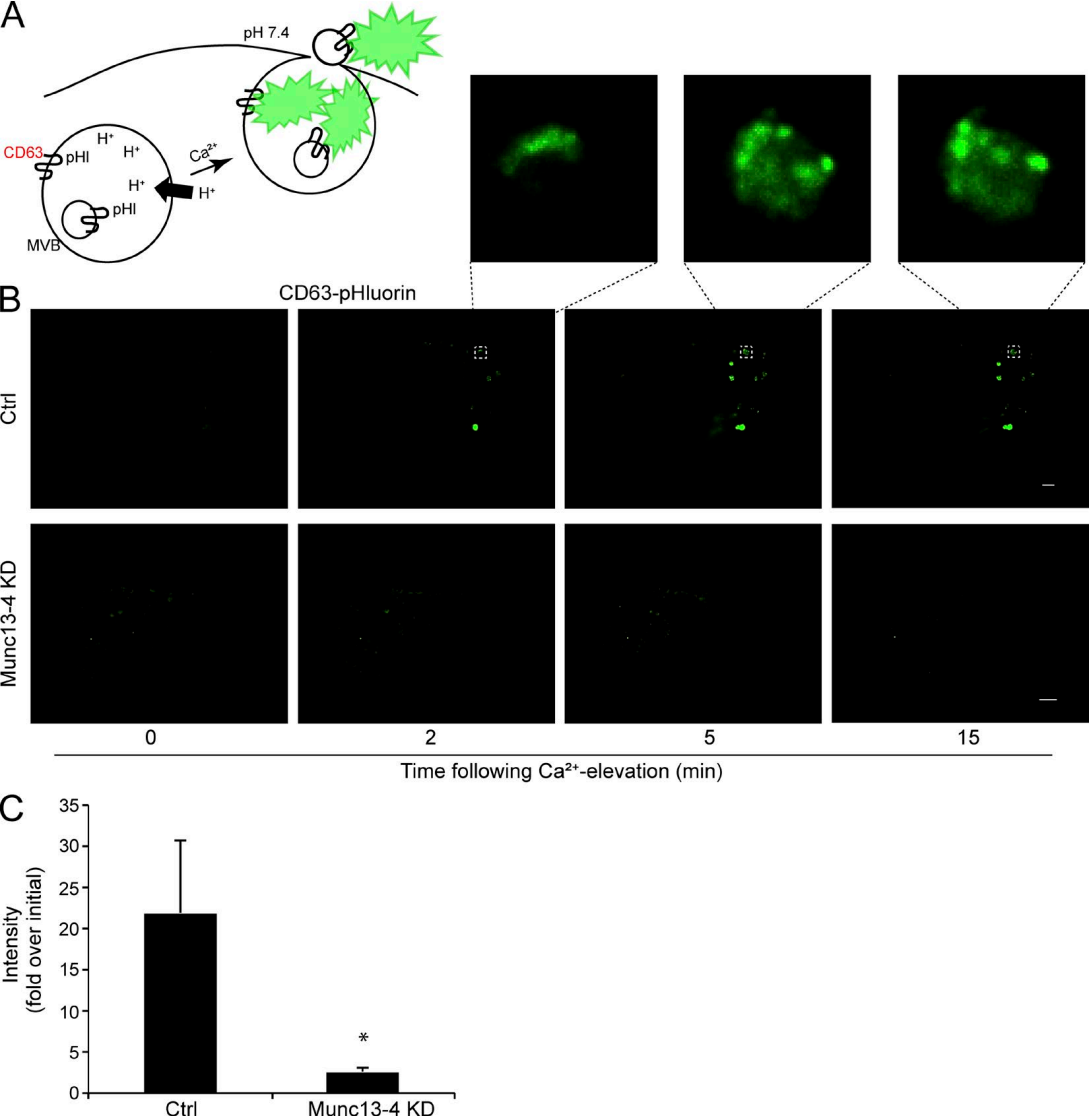
**Direct TIRF imaging of exosome secretion dependent on Munc13-4. (A)** pH-sensitive pHluorin (pHl) was inserted into loop 1 of CD63, which localizes to both the limiting membrane and ILVs of MVBs. Initially quenched at the low pH in the MVB, CD63-pHluorin increases fluorescence upon fusion of MVBs with the plasma membrane. **(B)** MDA-MB-231 cells stably expressing control shRNA (Ctrl, upper row) or Munc13-4 shRNA (Munc13-4 KD, lower row) were transfected with CD63-pHluorin plasmid, stimulated with 1.25 µM ionomycin (at zero time), and imaged for 15 min by TIRF microscopy. The surface of single cells is shown. In the upper row, ionomycin treatment elicited the release of brightened CD63-pHluorin–containing ILVs released into TIRF field. Bar, 5 µm. Inset shows one of the MVBs releasing ILVs as exosomes. Bar, 1 µm. See Videos 2 and 3. **(C)** Intensity of CD63-pHluorin signal is quantified as fold increase over initial fluorescent value reported as mean values ± SE (≥9 cells/group; *, P < 0.05).

Our studies indicate the presence of a Ca^2+^-sensitive secretory MVB that is the storage compartment for ILVs before exosome release. Although it is generally accepted that exosomes correspond to ILVs released from MVBs, there has been some uncertainty about whether vesicles budding from the plasma membrane also contribute to the release of exosome markers. Previous imaging studies by total internal reflection fluorescence (TIRF) microscopy in live cells focused on cellular migration rather than resolving ILV release (Sung et al., 2015). Because CD63 localizes to ILVs and the limiting MVB membrane (Fig. 3), we directly imaged MVB exocytosis and exosome release by TIRF microscopy using a CD63 construct with pH-sensitive pHluorin inserted into a luminal/extracellular loop (Fig. 6 A; Sung et al., 2015). At the low pH of the MVB, little fluorescence was observed by TIRF microscopy in MDA-MB-231 cells; however, after a 2–6-min delay, Ca^2+^-stimulation dramatically increased CD63-pHluorin fluorescence by 20-fold (Fig. 6 B, upper panel and insets; and Video 2). Exosomes were imaged as diffraction-limited fluorescent vesicles released into the TIRF field from the much larger MVBs (Fig. 6 B, insets). MVBs remained fused with the plasma membrane for >10 min, allowing multiple ILVs to be exposed at the cell surface, with some released into the medium. In contrast, Ca^2+^-stimulation of cells deficient in Munc13-4 showed no increase in CD63-pHluorin fluorescence (Fig. 6 B, lower panel; and Video 3) as summarized across multiple studies (Fig. 6 C). The results confirm that Munc13-4 is essential for Ca^2+^-stimulated exosome secretion.

### Munc13-4 regulates extracellular matrix degradation

**Figure 7.**
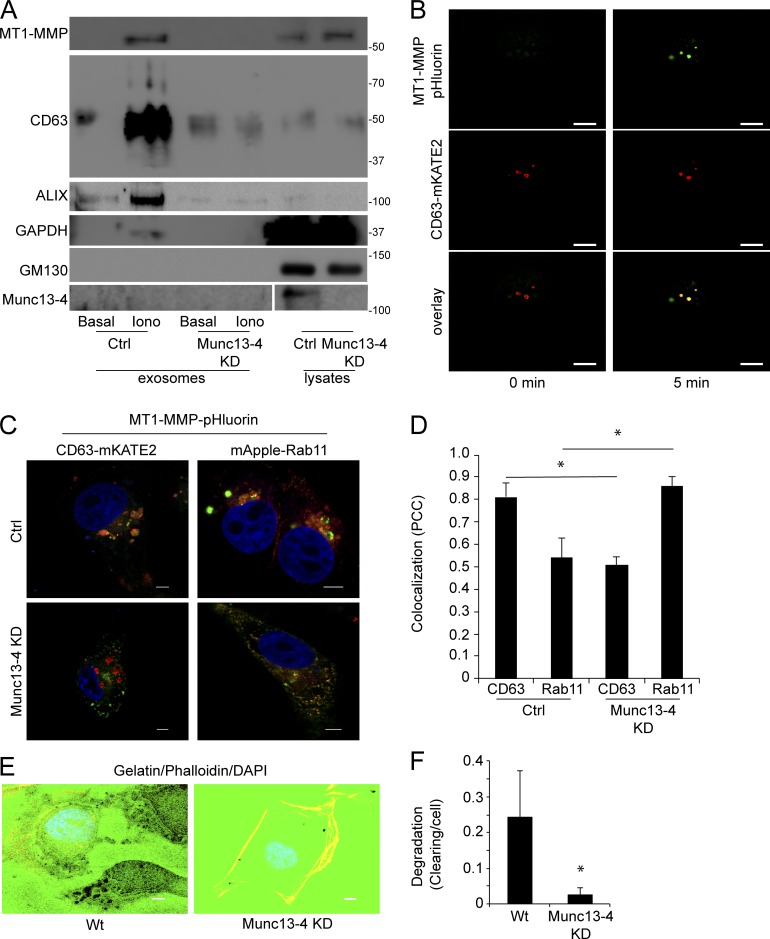
**ECM degradation by exosomal MT1-MMP is dependent on Munc13-4. (A)** Exosomes (100,000-*g* pellet) and 400 µg cell lysate were prepared from MDA-MB-231 cells stably expressing control shRNA (Ctrl) or Munc13-4 shRNA (Munc13-4 KD) and analyzed for indicated proteins by SDS-PAGE Western blotting. Exosomes secreted basally or from cells stimulated with 1.25 µM ionomycin for 1 h were compared. **(B)** MDA-MB-231 cells expressing CD63-mKATE2 and MT1-MMP-pHluorin were imaged by TIRF microscopy before (0 min) and after treatment with 1.25 µM ionomycin for 5 min. **(C)** Control or Munc13-4 KD cells expressing MT1-MMP-pHluorin and CD63-mKATE2 or mApple-Rab11a were fixed, permeabilized to brighten pHluorin, and imaged by confocal microscopy. **(D)** Pearson’s correlation coefficient for pHluorin versus mKATE2 or mApple is indicated as mean values ± SE (15 cells/group from three separate preparations are shown). **(E)** MDA-MB-231 cells stably expressing control shRNA (Ctrl) or Munc13-4 shRNA were grown on cover slides coated with Oregon-Green-Gelatin for 16 h and labeled with phalloidin and DAPI. Zoomed images from representative cells are shown. **(F)** Gelatin degradation was calculated as a percentage of clearing of gelatin from the larger field per cell, shown as mean values ± SE (nine fields of view [5–25 cells]/group from three separate preparations). *, P < 0.05. Bars, 5 μm.

Exosomes play numerous roles in cancer progression (de Gassart et al., 2004; Liu et al., 2006; Clayton et al., 2007, 2008; Bobrie et al., 2011; Filipazzi et al., 2012; Kharaziha et al., 2012; Hoshino et al., 2013; Sung et al., 2015; Fu et al., 2016; Kalluri, 2016; Sinha et al., 2016). Whole-genome sequencing of human tumors (TCGA cBioPortal) revealed amplification of Munc13-4 mRNA (UNC13D) in breast, pancreatic, and lung carcinomas in up to 28% of tumors, whereas other members of the Munc13 family showed no consistent up-regulation (Gao et al., 2013). During the early stages of metastasis, cancer cells degrade extracellular matrix and break free of the tumor mass to colonize additional tissues. Exosomes positive for membrane type 1 matrix metalloproteinase (MT1-MMP) are released from cancer cells to degrade the extracellular matrix, induce invadopodia, and initiate early stages of metastasis (Poincloux et al., 2009). We found that acute (1 h) Ca^2+^-stimulation of MDA-MB-231 cells caused a ∼10-fold increase in MT1-MMP^+^ exosome release, whereas the depletion of Munc13-4 reduced both basal and Ca^2+^-stimulated MT1-MMP^+^ exosome release (Fig. 7 A). MT1-MMP-pHluorin undergoing exocytosis in response to Ca^2+^ stimulation coincided with CD63^+^ structures, confirming that MT1-MMP is released on exosomes (Fig. 7 B and Video 4).

Munc13-4 KD cells exhibited an approximately threefold increase in cellular MT1-MMP levels (Fig. 7 A). MT1-MMP traverses the endosomal pathway and potentially traffics from Rab11^+^ endosomes to MVBs for exosome release. In fixed MDA-MB-231 cells, MT1-MMP-pHluorin primarily localized to CD63^+^ MVBs (Fig. 7 C), similar to the endogenous enzyme (not depicted). Overexpression of mApple-Rab11 resulted in a shift to a higher proportion of MT1-MMP colocalizing with Rab11 (Fig. 7 D). The depletion of Munc13-4 also increased MT1-MMP colocalization with Rab11^+^ endosomes and decreased MT1-MMP colocalization with CD63^+^ structures (Fig. 7 D). The results suggest that MT1-MMP traffics to MVBs by a Rab11/Munc13-4–dependent pathway. MDA-MB-231 cells were found to degrade fluorescent gelatin in an in vitro assay of ECM degradation to produce clearings at invadopodia (Fig. 7, E and F). Munc13-4 KD was found to substantially reduce gelatin degradation (Fig. 7 F) as well as the release of lysosomal hydrolases cathepsin B and β-hexosaminidase (not depicted) that also function in ECM degradation. Thus, Munc13-4 plays an essential role in the release of MT1-MMP–containing exosomes critical for early stages of cancer progression.

## Discussion

The key new findings in this study reveal the central importance of Munc13-4 for exosome release and identify Munc13-4’s essential role in generating Ca^2+^-sensitive secretory MVBs for exosome release. MVBs undergo maturation to secretory MVBs receiving input from Rab11^+^ endosomes (Fig. 5 F). GTP bound Rab11a, Munc13-4, and Ca^2+^ control the fusion of Rab11^+^ endosomes with MVBs, whereupon secretory MVBs enlarge and may gain competence for Ca^2+^-triggered exocytosis. Cancer cells exhibit increased Ca^2+^ levels (Yang et al., 2009; Feng et al., 2010; Prevarskaya et al., 2011; Sun et al., 2014; Didiasova et al., 2015), and we show that Ca^2+^-dependent Munc13-4 is a critical component for stimulated exosome release. Whole-genome sequencing data from human tumors suggest that Munc13-4 is elevated in breast, pancreatic, and lung carcinomas (Gao et al., 2013), and we confirmed this at the protein level for several tumor cell lines. Exosomes are extensively implicated in cancer progression (de Gassart et al., 2004; Liu et al., 2006; Clayton et al., 2007, 2008; Bobrie et al., 2011; Filipazzi et al., 2012; Kharaziha et al., 2012; Hoshino et al., 2013; Sung et al., 2015; Fu et al., 2016; Kalluri, 2016; Sinha et al., 2016), and we found that Munc13-4 KD strongly inhibited exosome secretion and prevented extracellular matrix degradation as a marker for early-stage metastasis. The results suggest that Munc13-4 is a potential target for cancer treatment.

The role of Rab27a as a regulator of exosome release was confirmed in our studies in MDA-MB-231 cells. In screening for Rab27a effector proteins for basal exosome secretion in HeLa cells, Ostrowski et al. (2010) identified Slp4a and Slac2 but not Munc13-4 as possible Rab27 effectors. Rab27a in HeLa cells appears to act a late stage in exosome secretion involving MVB docking near the plasma membrane (Ostrowski et al., 2010). Although Munc13-4 functions as a Rab27 effector in some cells, Munc13-4 interacts with multiple Rab proteins including Rab11 (Higashio et al., 2016; Johnson et al., 2016; Woo et al., 2017). Our studies in MDA-MB-231 cells suggest that Munc13-4 functions as a Rab11a effector that operates in MVB maturation before MVB docking and fusion at the plasma membrane.

CD63 has become the gold standard to distinguish exosomes from other extracellular vesicles (Bobrie et al., 2012a; Kowal et al., 2014, 2016; Tkach and Théry, 2016) and was used as the primary exosome marker in this study. We used a quantitative filter blotting assay to monitor exosome secretion and reveal the stimulatory effect of Ca^2+^ and the dependence on Munc13-4 for exosome release. These findings were validated by a repertoire of standard exosome characterization methods (Fig. S1). Moreover, we directly imaged the Ca^2+^-stimulated release of CD63^+^ ILVs by TIRF microscopy and confirmed its dependence on Munc13-4. The KD of Munc13-4 also significantly reduced basal CD63^+^ exosome release but had only limited effects on basal CD9^+^ vesicle release. CD9 is present on nonexosomal extracellular vesicles (Bobrie et al., 2012a; Kowal et al., 2016), and it is likely that these account for basal CD9 release. Indeed, similar to a recent study (Jackson et al., 2017), we found that CD9 was present on basally released extracellular vesicles whose density differed from that of CD63^+^ exosomes. However, there was strong enhancement of CD9 on true exosomes released by Ca^2+^ stimulation (Fig. S1 B). Consistent with this, we observed that CD9 translocates from its primary plasma membrane localization in MDA-MB-231 cells to CD63^+^ MVBs after Ca^2+^ stimulation (Fig. S3). Our results show that Munc13-4 is required for basal and Ca^2+^-stimulated CD63^+^ exosome release as well as for Ca^2+^-stimulated CD9^+^ exosome release.

Aberrant Ca^2+^-signaling has long been associated with cancer progression, in which several Ca^2+^ channels are inappropriately elevated or expressed. Blocking Ca^2+^-channels severely limits metastatic potential, although the mechanisms are unclear (Lee et al., 2011; Prevarskaya et al., 2011; Monteith et al., 2012). Exosomes have been extensively implicated in cancer progression (de Gassart et al., 2004; Liu et al., 2006; Clayton et al., 2007, 2008; Bobrie et al., 2011; Filipazzi et al., 2012; Kharaziha et al., 2012; Hoshino et al., 2013; Sung et al., 2015; Fu et al., 2016; Kalluri, 2016; Sinha et al., 2016), and the Ca^2+^ stimulation of exosome release was previously reported (Vincent-Schneider et al., 2001; Savina et al., 2002, 2003, 2005; Fauré et al., 2006). In our studies, Ca^2+^ binding–deficient Munc13-4 failed to rescue acute Ca^2+^-stimulated CD63^+^ exosome release in Munc13-4 KD cells, indicating that Munc13-4 serves as an essential Ca^2+^ sensor. We did not detect Munc13-4 or Rab11a at the sites of exosome release in TIRF microscopy (unpublished data), which suggests that Munc13-4 and Rab11 do not directly regulate MVB–plasma membrane fusion in MDA-MB-231 cells. Rather, the Ca^2+^- and Munc13-4–dependent fusion of Rab11^+^ endosomes with MVBs may mediate the transfer to MVBs of proteins that confer competence for Ca^2+^-triggered fusion, which might be Slp4a or synaptotagmin-7 (Ostrowski et al., 2010; Hoshino et al., 2013). Collectively, our results suggest that Munc13-4’s role in resting cancer cells involves endosomal remodeling to generate secretion-competent MVBs using Ca^2+^ increases originating either from the endosomal structures (Ghislat and Knecht, 2013; He et al., 2016) or from increased cytosolic Ca^2+^ from Orai1 Ca^2+^ channels that are essential for metastasis (Yang et al., 2009; Feng et al., 2010) and required for enhanced exosome release (Didiasova et al., 2015). Our studies indicate that Ca^2+^ binding to Munc13-4 is essential for its activity in generating secretion-competent MVBs.

Secretory MVBs apparently constitute a small pool of CD63^+^ MVBs, as assessed by quantitating percentage CD63^+^ exosome release. It is possible to compare our exosome release estimates of Fig. 1 with the exosome exposure and release of CD63-pHluorin from MVBs detected by TIRF microscopy in Fig. 6. For the latter, we found 13 ± 6 CD63-pHluorin structures brightening in the TIRF field after Ca^2+^ stimulation. This was from a total 63 ± 24 CD63-pHluorin^+^ structures per cell, indicating that ∼20% of CD63^+^ MVBs undergo exocytosis. Converting this into fractional CD63^+^ exosome release requires two other considerations. First, exosomes may be tethered to membrane, reducing by approximately fourfold their release into the medium (Edgar et al., 2016). In addition, EM studies have shown that CD63 is on ILVs as well as on the MVB-limiting membrane, so ∼60% of the CD63 may be on exosomes (Escola et al., 1998). Taking these factors into consideration would convert the estimate of ∼20% MVB exocytosis into ∼3% exosome release. In the filter binding assay of Fig. 1, we detect ∼1% exosome release, but similar corrections (fourfold underestimation of exosomes released into medium coupled with 60% of CD63 in MVBs on ILVs) would bring this estimate to ∼6% exosome release. Thus, these corrections bring the two assays close to congruence, indicating that a small percentage of CD63^+^ ILVs are released as exosomes in our assays.

In MDA-MB-231 cells, little Rab11a and Munc13-4 was found on CD63^+^ MVBs even in the presence of Ca^2+^, yet both regulated the size of MVBs, suggesting their role in generating secretion-competent MVBs. Expression of the GTP-locked dominant active Rab11a resulted in greater colocalization of Rab11a and Munc13-4 with CD63, indicating a trafficking role from the Rab11^+^ endosome to the MVB. Previously, it was found that Munc13-4 is required for the overall fusion of cytotoxic granules with the plasma membrane in CTLs (Feldmann et al., 2003), where its primary role may be in the fusion of recycling with late endosomes to generate the lysosome-related secretory organelle. Munc13-4 is not present on the cytotoxic granules in CTLs (Ménager et al., 2007) and NK cells (Wood et al., 2009) under resting conditions. Rab11^+^ endosomes and lytic granule precursors may fuse only transiently, as indicated by electron microscopy in resting cells (Ménager et al., 2007). Similarly, kiss-and-run fusion between Rab11^+^ endosomes and MVBs may occur in MDA-MB-231 cells without the net transfer of Rab11 or Munc13-4 to the MVBs. Exosomes were identified in reticulocytes (Harding et al., 1983; Johnstone et al., 1987), where 25–50% of loaded transferrin was lost in exosomes as reticulocytes matured to erythrocytes, indicating an important role for the recycling endosome in exosome release. In K562 cells, GFP-Rab11a was found on MVBs, and the major phenotype observed after manipulation of Rab11 was a change in size of MVBs. MT1-MMP traffics through both the Rab11^+^ recycling endosome and the MVB before release on exosomes (Poincloux et al., 2009; Hoshino et al., 2013; Sung et al., 2015). In control cells, MT1-MMP localizes to CD63^+^ MVBs; however, reduction of Munc13-4 shifts localization to Rab11^+^ endosomes and ablates MT1-MMP^+^ exosome release, suggesting that recycling endosome trafficking to the MVB plays an important biologically relevant role (see Fig. 5 F). In addition, as mutations of Munc13-4 lead to FHL3, it may be important to consider exosomes in the progression of this disease. Collectively, our results indicate that Munc13-4 acts as a Rab11a effector to generate secretory-competent, Ca^2+^-sensitive MVBs. The major finding is that Munc13-4 is an essential regulator of the enhanced Ca^2+^-stimulated exosome release in metastatic cells, which extends recent findings on the role of Munc13-4 in endosome remodeling (Ménager et al., 2007; He et al., 2016; Woo et al., 2017) and implies that Munc13-4 could be an important target for intervention in metastasis.

## Materials and methods

### Cells and materials

MDA-MB-231 cells were a gift from R. Anderson (University of Wisconsin-Madison, Madison, WI) and were cultured in DMEM (Life Technologies) supplemented with 10% FBS and antibiotics-antimycotics (Life Technologies). Panc-1 cells were a gift from J. Koa (University of Wisconsin-Madison, Madison, WI) and were cultured in DMEM supplemented with 10% FBS and antibiotics-antimycotics. A549 cells were a gift from J.E. Gern (University of Wisconsin-Madison) and were cultured in F12 medium supplemented with 10% FBS and antibiotics-antimycotics. 293FT cells (Life Technologies) used for lentivirus production were cultured in DMEM supplemented with 10% FBS, antibiotics-antimycotics, and 500 µg/ml Geneticin (Life Technologies). For adenovirus production, AD-293 cells were purchased from Agilent and were cultured in DMEM supplemented with sodium pyruvate (Life Technologies), 10% FBS, and antibiotics-antimycotics. Cells were maintained at 37°C in a 5% CO_2_ atmosphere. Cell lines are routinely tested for mycoplasma contamination using Mycoprobe Mycoplasma Detection kit (R&D Systems). Lentiviral shRNA constructs used were as follows: for human Rab27a, 5′-GTGCGATCAAATGGTCATGCC-3′; for human Rab11a, 5′-GCAACAATGTGGTTCCTAT-3′; and for human Munc13-4, 5′-CATCAGCGGTGGATCTATC-3′. pLKO.1 scramble (control) shRNA was a gift from D. Sabatini (Whitehead Institute, Cambridge, MA; plasmid 1864; Addgene). pLKO.1-TRC cloning vector was a gift from D. Root (Broad Institute, Cambridge, MA; plasmid 10878; Addgene). psPAX2 was a gift from D. Trono (plasmid 12260; Addgene). pMD2.G was a gift from D. Trono (École polytechnique fédérale de Lausanne, Lausanne, Switzerland; plasmid 12259; Addgene). Lentiviruses were produced as previously described (Woo et al., 2017). Wild-type GFP-Munc13-4 or mutants (D127N, D133N in C2A [Munc13-4 C2A*] and D941N, D947N in C2B [Munc13-4 C2B*]) were previously generated (Wood et al., 2009). mApple-Rab11a-7 was a gift from M. Davidson (plasmid 54942; Addgene). CD63-mKATE2 was generated by cloning human CD63 purchased from Origene into pmKATE2-N (Evrogen). CD63 with pHluorin inserted into extracellular loop 1 and MT1-MMP-pHluorin were gifts from A. Weaver (Vanderbilt University, Nashville, TN; Hoshino et al., 2013; Sung et al., 2015). GFP-Rab11a and mutant Q70L and S25N adenoviruses were gifts from R. Kuruvilla (Johns Hopkins University, Baltimore, MD). Myc-Munc13-4 lentivirus was purchased from Cyagen. shRNA-resistant Myc-Munc13-4 and mutants (C2A* and C2B*) were generated by site-directed mutagenesis using QuikChange II XL (Agilent). The mCherry-Rab7 construct was a gift from A. Sorkin (University of Pittsburgh, Pittsburgh, PA).

Antibodies used were rabbit monoclonal Munc13-4 (residue 20–80; EPR4914) from Abcam and goat polyclonal Munc13-4 antibody (residue 967–980; NB100-41385) from Novus Biologicals. Rabbit polyclonal Munc13-4 C2A antibody was provided by H. Horiuchi (Tohoku University, Sendai, Japan). Rabbit monoclonal anti-Rab27a (EPR3021) was purchased from Abcam. These antibodies were characterized for immunofluorescence and immunoblotting by comparing wild-type cells with KD. Rabbit polyclonal anti-ALIX (ABC40) was purchased from Millipore, rabbit monoclonal anti-CD9 (EPR2949) from Abcam, and monoclonal CD63 (H5C6), CD81 (JS-81), and GM130 (35) antibodies from BD Biosciences. Rabbit monoclonal LAMP1 (D2D11) was purchased from Cell Signaling Technologies. Alexa fluorophore-conjugated secondary antibodies were from Molecular Probes. All other chemicals were purchased from Sigma.

### Immunoblotting

Cells were washed and solubilized with 0.1% Triton X-100, 10 mM Tris-Cl, pH 8.0, and 1 mM EDTA in the presence of a protease inhibitor cocktail and 1 mM phenylmethanesulfonyl fluoride for 10 min at 4°C with gentle rocking. SDS was added to 2% final concentration and boiled for 5 min at 96°C. Protein concentration was determined by BCA assay kit (Pierce Chemical). 200–400 µg of lysate protein was mixed with 4× SDS sample buffer (250 mM Tris-HCl, pH 6.8, 30% glycerol, 300 mM dithiothreitol, 8% SDS, and 0.02% bromophenol blue) and boiled for 5 min at 96°C before loading onto 8–15% polyacrylamide gels. After electrophoresis (Bio-Rad), proteins in the gel were electrotransferred to nitrocellulose membranes (0.45-µm pore; Bio-Rad). Membranes were blocked with 5% skim milk in PBS with 0.1% Tween 20 (PBST) for 1 h at room temperature and incubated with primary antibodies in 1% BSA in PBST overnight. Primary antibody was detected by incubation with HRP-conjugated secondary antibodies for 1 h at RT. Blots were developed by enhanced chemiluminescence kit (Pierce) and imaged by ImageQuant LAS 4000 system (GE Healthcare) in the ultra mode (16 × 16 bin). Band intensity was quantified using ImageJ (National Institutes of Health), and images were cropped with brightness and contrast adjusted with the “auto” function. TIFF files were enlarged twice with separate bicubic smoothing and adjusted with the “levels” function in Photoshop so that the black point and white point slider encompassed the signal in the image histogram, thereby removing the lack of signal in the remainder of the image histogram. The midtone slider was adjusted to increase the intensity of the band of the protein of interest.

### Transfection and immunofluorescence

For fluorescence microscopy, glass coverslips were coated with 0.1 mg/ml poly-d-lysine at 37°C (Sigma-Aldrich). To express fluorescence-tagged proteins, 4 µg DNA was mixed with Lipofectamine 2000 (Thermo Fisher) as indicated in the manufacturer’s instructions. Media were replaced after 6 h, and constructs typically expressed fluorescence proteins within 48-h incubation. For stimulation, cells were incubated in DMEM free of phenol red with 10 mM CaCl_2_ in the presence or absence of 2.5 µM ionomycin for 20–120 min. For live-cell imaging, glass-bottom culture dishes (MatTek) were coated with poly-d-lysine (0.1 mg/ml) at 37°C for 30 min. For immunostaining, cells were washed and fixed in 4% formaldehyde in PBS for 8 min at room temperature and permeabilized with 0.2% Triton X-100 in PBS for 10 min at room temperature. Nonspecific binding was blocked by incubating with 5% goat or donkey serum for 1 h. The cells were incubated with primary antibodies diluted in the blocking solution overnight at 4°C. The cells were washed and incubated with 20 µg/ml Alexa Fluor–conjugated secondary antibodies for 1 h at room temperature. After additional washing, nuclei were stained with 2 µg/ml Hoechst 33342 for 5 min at room temperature. Cells attached to glass coverslips were buffered by Slowfade Gold solution (Life Technologies) and mounted on glass slides.

### Fluorescence microscopy and image analysis

Cells were imaged by TIRF illumination using a Nikon Eclipse Ti microscope controlled by NIS element software. Images were captured with an iXon Ultra EM-CCD camera through a Nikon APO 100× TIRF NA-1.49 objective. Time-lapse imaging for live cells was conducted in a humidified imaging chamber maintaining 37°C (Tokai Hit). SIM images were acquired with a Nikon N-SIM microscope (SR-APO TIRF NA-1.49 100× lens, Andor iXon 3 EM-CCD camera, 408-, 488-, 561-, 640-nm laser lines) using 3D-SIM mode and reconstructed with NIS software. Confocal images were acquired with a Nikon A1-R confocal system using GaAsP detectors. TIRF and confocal images were deconvolved, processed, and analyzed by NIS software (Nikon). Deconvolution artifacts were checked by linescan analysis comparing with original images. For fluorescence overlap analysis, Manders’ overlap coefficient was calculated by JACoP plugin in ImageJ for fluorescent pixels above threshold (Bolte and Cordelières, 2006). Fluorescence channels were typically arranged as red fluorescence, channel 1; green fluorescence, channel 2 in JACoP plugin. Manders’ coefficients produced by JACoP included M1 and M2: M1, fraction of channel 1 overlapping with channel 2; M2, fraction of channel 2 overlapping with channel 1. Channel arrangements are indicated in figure legends. For vesicle profile area determination, images were binarized by threshold function, and circular objects were detected by particle analysis function in ImageJ. Oregon-green gelatin degradation assays were calculated as amount of clearing of gelatin normalized to cell number, as previously described (Berdeaux et al., 2004).

### Exosome purification

Exosomes were purified using standard methods to establish the identity of exosomes (Lötvall et al., 2014). MDA-MB-231 cells were plated at 50–70% confluence 18 h before exosome collection. Cells were extensively washed in PBS buffer + 100 µM EDTA. Exosomes were collected in DMEM free of phenol red with 10 mM CaCl_2_ and 0.1% BSA. For exosome collection, cells were left untreated or were stimulated with 2.5 µM ionomycin for 120 min. Stimulation was limited to <4 h, as the Golgi membrane marker GM130 was detected in the exosome fraction at longer times. Supernatants were cleared of cellular debris and large extracellular membranes by centrifugation at 300 *g* for 5 min followed by 1,000 *g* for 10 min and twice at 10,000 *g* for 10 min in the JA-10 rotor. Supernatants were centrifuged in a Ti70 rotor at 100,000 *g* for 120 min. The pellet was resuspended, washed in PBS, and centrifuged for 120 min at 100,000 *g*. The 100,000-*g* pellet was analyzed by Philips TM10 transmission electron microscopy, Wyatt Möbius dynamic light scattering, and SYPRO Ruby staining (Invitrogen). The 100,000-*g* pellet was further centrifuged at 150,000 *g* for 16 h in a SW41 rotor, and purified exosomes were collected at the 20–40% sucrose interface as described previously (Shurtleff et al., 2016) or the 100,000-*g* pellet was layered on a stepwise sucrose gradient as described previously (Raposo et al., 1996; Théry et al., 1999). Exosomes were vacuum filtered onto nitrocellulose membrane using a Slot-Blot apparatus (Schleicher & Schuell). Exosomes were lysed in 4× SDS sample buffer in the presence (or absence for CD63) of DTT. Protein was detected using well-described commercially available antibodies listed above.

### Quantitative exosome release assay

Cells were seeded 18 h before exosome collection to reach a density of 70% confluence with cell–cell contacts minimized. Cells were washed 3× in PBS + 100 µM EDTA before collection of exosomes in DMEM free of phenol red with 10 mM CaCl_2_. Stimulation time with 2.5 µM ionomycin was limited to 20–60 min, as times longer than 4 h resulted in detection of the Golgi marker GM130 in the exosome fraction, indicating cellular death. Exosome-containing media was centrifuged at 300 *g* for 5 min, at 1,000 *g* for 10 min, and at 10,000 *g* for 10 min to provide supernatants for filtering onto nitrocellulose membrane using a Slot-Blot apparatus (Schleicher & Schuell). Known exosomes markers were detected by well-described commercially available antibodies listed above. Total cellular material was acquired and loaded on membrane to allow for quantification of exosome release as a percentage of total cellular material. After chemiluminescence development, slot blots were imaged with an ImageQuant LAS 4000 unit ordinarily in the ultra (16 × 16) bin mode to maximize sensitivity for detecting low-abundance antigens. Binning in the ultra mode increases signal/noise and decreases resolution but preserves quantitative information. Images were saved as TIFF files and quantified in ImageJ. For presentation, images were adjusted for brightness and contrast with the “auto” function of ImageJ, enlarged twice with bicubic smoothing, and adjusted with the “levels” function in Photoshop so that the black point and white point slider were centered on the signal in the image histogram. The midtone slider was adjusted to increase the intensity of the protein band, and the image was cropped and imported into the figure.

### Online supplemental material

Fig. S1 shows characterization of Ca^2+^- and Munc13-4–dependent released exosomes. Fig. S2 shows that Panc-1 cells exhibit Ca^2+^- and Munc13-4–dependent exosome release. Fig. S3 shows that CD9 translocates to CD63^+^ MVBs after Ca^2+^ elevation. Video 1 shows Ca^2+^-induced membrane translocation of Munc13-4 in MDA-MB-231 cells. Video 2 shows live cell imaging of exosome release in MDA-MB-231 cells. Video 3 shows live cell imaging of exosome release in MDA-MB-231 cells. Video 4 shows live cell imaging of exosomal MT1-MMP release.

## Supplementary Material

Supplemental Materials (PDF)
