# Correction: A Ca^2+^-stimulated exosome release pathway in cancer cells is regulated by Munc13-4

**DOI:** 10.1083/jcb.20171013203042019c

**Published:** 2019-03-09

**Authors:** Scott W. Messenger, Sang Su Woo, Zhongze Sun, Thomas F.J. Martin

Vol. 217, No. 8, August 6, 2018. 10.1083/jcb.201710132.

The top blot shown in Fig. S1 A was mistakenly inverted prior to publication. The corrected Fig. S1 is shown below. The text and figure legends were correct as originally described. This correction does not affect the conclusions of the paper.

The supplemental PDF has been corrected. These errors appear only in PDF versions downloaded on or before March 8, 2019.

**Figure S1.**
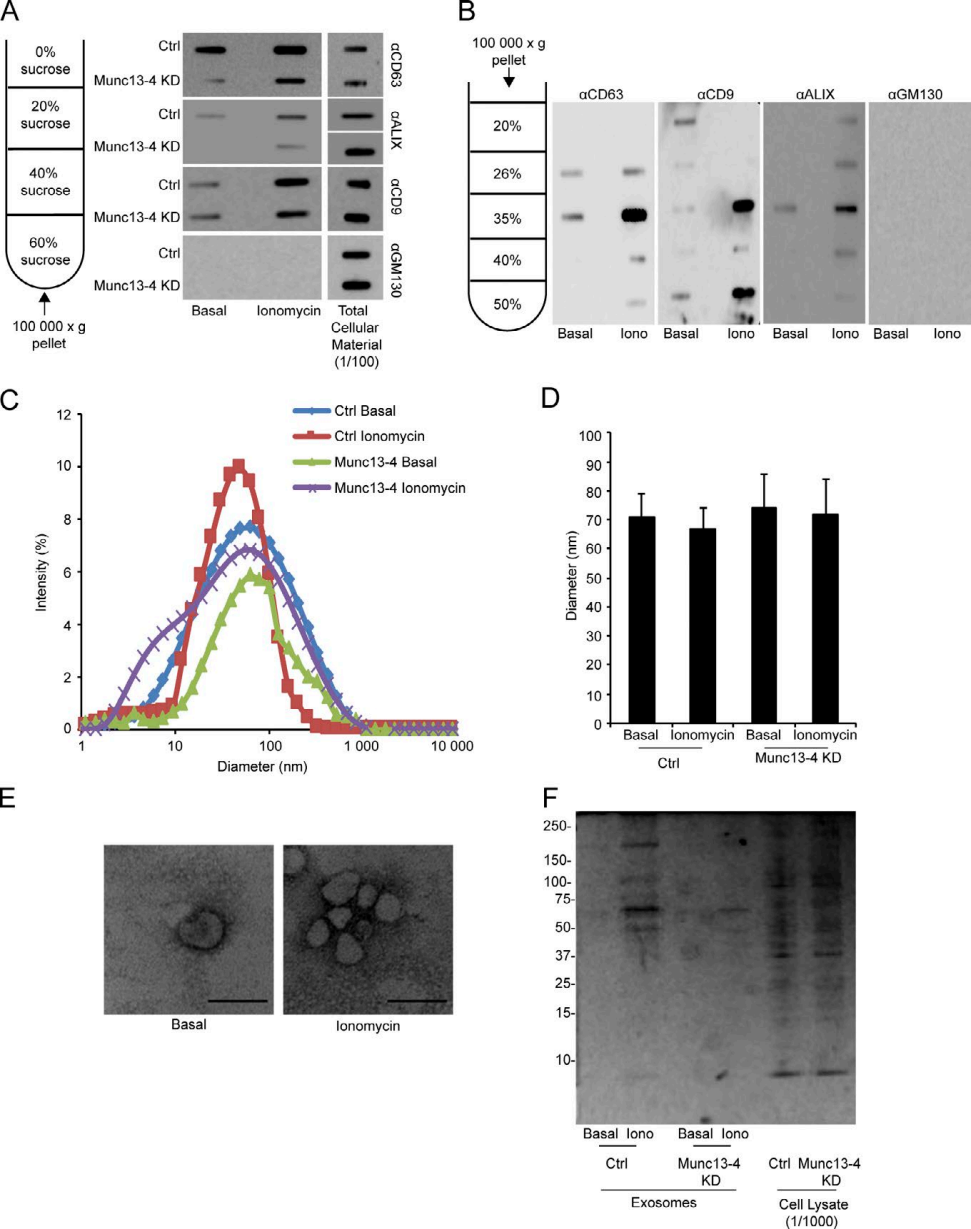
**Characterization of Ca^2+^- and Munc13-4–dependent released exosomes. (A)** Culture media supernatants (as in Fig. 1 B) from untreated or ionomycin-treated MDA-MB-231 cells were pelleted at 100,000 *g*, floated to the 20%/40% interface of a sucrose block gradient as in Shurtleff et al. (2016), filtered onto nitrocellulose, and immunoblotted for CD63, ALIX, CD9, and GM130. 1% of total cellular lysate was similarly filtered. **(B)** The 100,000-*g* pellet fractions from untreated or ionomycin-treated cells were purified on a density block gradient as in Raposo et al. (1996) and Théry et al. (1999) to 1.19 g/ml density, filtered onto nitrocellulose, and immunoblotted for CD63, CD9, ALIX, and GM130. **(C)** 100,000-*g* pellet fractions from the media of MDA-MB-231 cells (with indicated treatments) were analyzed by dynamic light scattering for size distribution. **(D)** The mean diameter determined by dynamic light scattering is shown as mean values ± SE for n = 6. **(E)** 100,000-*g* pellet fractions from control (Ctrl) or Munc13-4 KD MDA-MB-231 cells were imaged by electron microscopy. Bar, 100 nm. **(F)** 100,000-*g* pellet fractions from control or ionomycin-treated MDA-MB-231 cells or Munc13-4 KD cells were analyzed by SDS-PAGE and stained with SYP​RO Ruby. 0.1% cell lysates were similarly analyzed. Approximate molecular mass markers in kilodaltons are indicated.

