# Supplementary material for: A Ca2+-stimulated exosome release pathway in cancer cells is regulated by Munc13-4
Source: J Cell Biol. 2018 Aug 6;217(8):2877–90. doi: 10.1083/jcb.201710132 (PMC6080937; doi:10.1083/jcb.201710132)
Supplement: Supplemental Materials (PDF) [file JCB_201710132_sm.pdf]

Supplemental material

Messenger et al., https://doi.org/10.1083/jcb.201710132

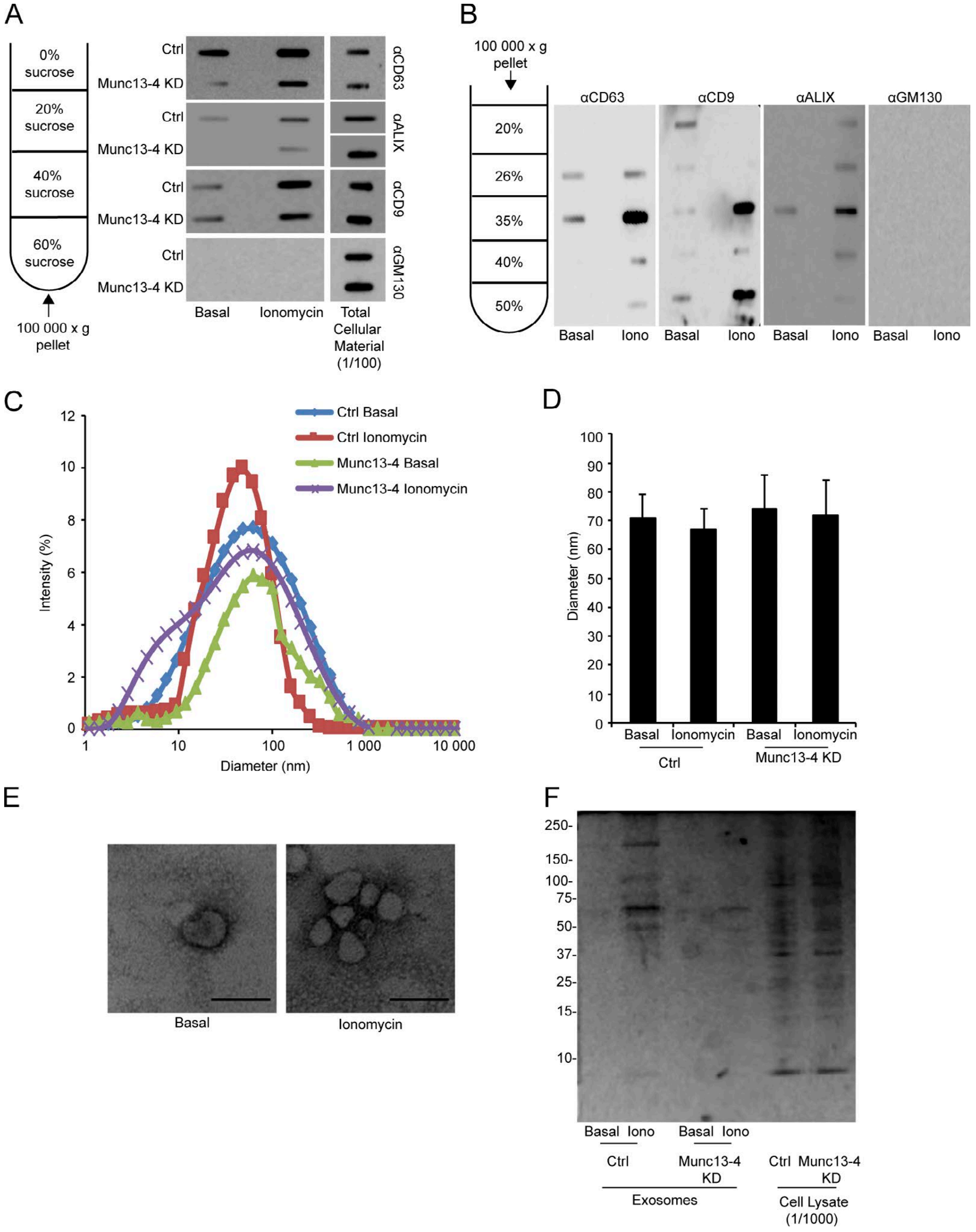

Figure S1. **Characterization of Ca^{2+}- and Munc13-4–dependent released exosomes. (A)** Culture media supernatants (as in Fig. 1 B) from untreated or ionomycin-treated MDA-MB-231 cells were pelleted at 100,000 *g*, floated to the 20%/40% interface of a sucrose block gradient as in Shurtleff et al. (2016), filtered onto nitrocellulose, and immunoblotted for CD63, ALIX, CD9, and GM130. 1% of total cellular lysate was similarly filtered. **(B)** The 100,000-*g* pellet fractions from untreated or ionomycin-treated cells were purified on a density block gradient as in Raposo et al. (1996) and Théry et al. (1999) to 1.19 g/ml density, filtered onto nitrocellulose, and immunoblotted for CD63, CD9, ALIX, and GM130. **(C)** 100,000-*g* pellet fractions from the media of MDA-MB-231 cells (with indicated treatments) were analyzed by dynamic light scattering for size distribution. **(D)** The mean diameter determined by dynamic light scattering is shown as mean values ± SE for $n = 6$. **(E)** 100,000-*g* pellet fractions from control (Ctrl) or Munc13-4 KD MDA-MB-231 cells were imaged by electron microscopy. Bar, 100 nm. **(F)** 100,000-*g* pellet fractions from control or ionomycin-treated MDA-MB-231 cells or Munc13-4 KD cells were analyzed by SDS-PAGE and stained with SYPRO Ruby. 0.1% cell lysates were similarly analyzed. Approximate molecular mass markers in kilodaltons are indicated.

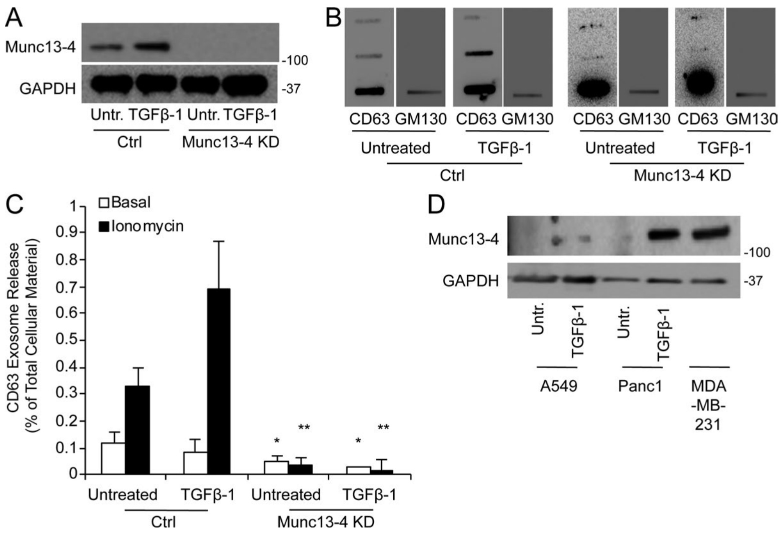

Figure S2. **Panc-1 cells exhibit Ca^{2+}- and Munc13-4–dependent exosome release. (A)** Panc-1 cells stably expressing shRNA for scrambled (Ctrl) or Munc13-4 were left untreated (Untr) or were treated with TGFβ-1 for 24 h. **(B)** Supernatants from Panc-1 cells either untreated or stimulated with 1.25 µM ionomycin for 30 min were centrifuged at 1,000 *g* to remove cellular debris and 10,000 *g* to remove large extracellular vesicles. The resulting supernatant was filtered onto membrane and analyzed for protein content. **(C)** Quantification of CD63 exosome release as percentage of total from B is shown as mean values ± SE (n = 5; *, $P < 0.05$; **, $P < 0.01$). **(D)** Lysates from untreated or TGFβ-1–treated A549 and Panc-1 cells and from untreated MDA-MB-231 cells were analyzed by SDS-PAGE and Western blotting for Munc13-4 and GAPDH.

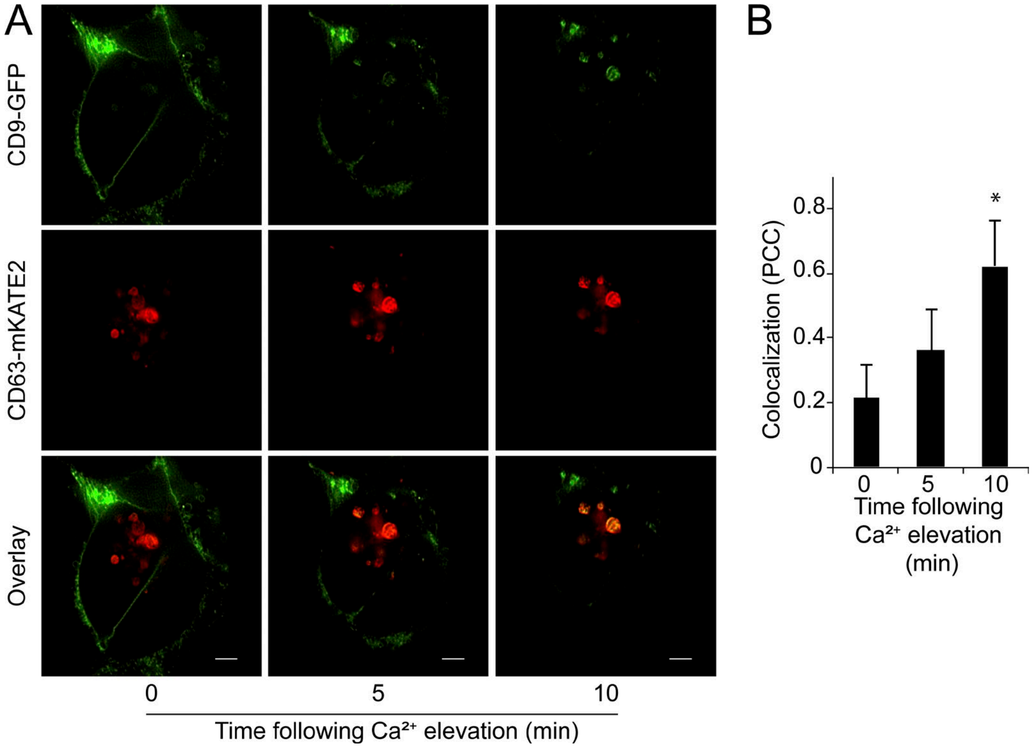

Figure S3. **CD9 translocates to $CD63^{+}$ MVBs after Ca^{2+}-elevation. (A)** MDA-MB-231 cells expressing CD9-GFP and CD63-mKATE2 were imaged by epifluorescence microscopy before (0 min) and after treatment with 1.25 µM ionomycin for 5 and 10 min. **(B)** Pearson's correlation coefficient for CD9-GFP versus CD63 immunolocalization. Mean values ± SE (nine cells/group from three separate preparations) are shown. *, $P < 0.05$.

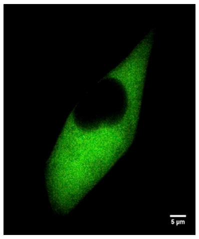

Video 1. **Ca^{2+}-induced membrane translocation of Munc13-4 in MDA-MB-231 cells.** Live-cell imaging (every 30 s in wide field) of GFP-Munc13-4 was conducted in MDA-MB-231 cells stimulated with 1.25 µM ionomycin for 30 min.

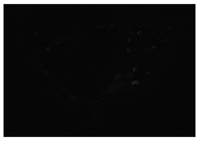

Video 2. **Live-cell imaging of exosome release in MDA-MB-231 cells.** MDA-MB-231 cells stably expressing control shRNA were transfected with CD63-pHluorin plasmid, stimulated with 1.25 µM ionomycin, and imaged every 30 s by TIRF microscopy for 20 min.

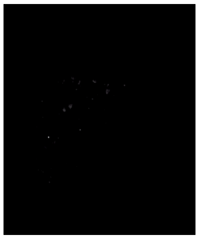

Video 3. **Live-cell imaging of exosome release in MDA-MB-231 cells.** MDA-MB-231 cells stably expressing Munc13-4 shRNA were transfected with CD63-pHluorin plasmid, stimulated with 1.25 µM ionomycin, and imaged every 30 s by TIRF microscopy for 20 min.

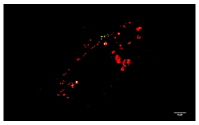

Video 4. **Live-cell imaging of exosomal MT1-MMP release.** MDA-MB-231 cells expressing CD63-mKate2 and MT1-MMP-pHluorin were stimulated with 1.25 µM ionomycin and imaged every 15 s by TIRF microscopy for 15 min.

References

Raposo, G., H.W. Nijman, W. Stoorvogel, R. Liejendekker, C.V. Harding, C.J. Melief, and H.J. Geuze. 1996. B lymphocytes secrete antigen-presenting vesicles. *J. Exp. Med.* 183:1161–1172. https://doi.org/10.1084/jem.183.3.1161

Shurtleff, M.J., M.M. Temoche-Diaz, K.V. Karfilis, S. Ri, and R. Schekman. 2016. Y-box protein 1 is required to sort microRNAs into exosomes in cells and in a cell-free reaction. *eLife.* 5:e19276. https://doi.org/10.7554/eLife.19276

Théry, C., A. Regnault, J. Garin, J. Wolfers, L. Zitvogel, P. Ricciardi-Castagnoli, G. Raposo, and S. Amigorena. 1999. Molecular characterization of dendritic cell-derived exosomes. *J. Cell Biol.* 147:599–610. https://doi.org/10.1083/jcb.147.3.599
